# Treatment Burden and Mobile Phone Use Among Adults With Human Immunodeficiency Virus (HIV) and Type 2 Diabetes: A Qualitative Formative Study

**DOI:** 10.7759/cureus.109888

**Published:** 2026-05-29

**Authors:** Kizza Gerald, Angella Musiimenta, Natumanya Deborah, Kimera Richard, William Wasswa, Wilson Tumuhimbise

**Affiliations:** 1 Computer Science, Mbarara University of Science and Technology, Mbarara, UGA; 2 Information Technology, Mbarara University of Science and Technology, Mbarara, UGA; 3 Biomedical Sciences and Engineering, Mbarara University of Science and Technology, Mbarara, UGA

**Keywords:** digital health, hiv, medication management, mobile phones, social support, treatment burden, type 2 diabetes, uganda

## Abstract

Background: Adults living with both human immunodeficiency virus (HIV) and type 2 diabetes in Uganda must manage multiple chronic conditions under circumstances of constrained income, fragmented care, and variable social support. However, there is limited qualitative evidence on how treatment burden is experienced in everyday life, how mobile phones are already being used to cope with that burden, and what this implies for future intervention design.

Objective: This study aimed to explore treatment burden among adults living with HIV and type 2 diabetes in Uganda, examine how patients informally use mobile phones and social support to manage medication and care, and identify user-informed requirements for a phone-based medication support intervention.

Methods: The study employed a qualitative design using in-depth interviews and focus group discussions (FGDs) in southwestern Uganda. Data were generated from 64 participants: 9 adults living with HIV and type 2 diabetes participated in individual in-depth interviews, and 55 participants took part in 11 FGDs. The FGDs included six patient FGDs involving 30 adults living with HIV and type 2 diabetes, four social supporter FGDs involving 20 nominated supporters, and one healthcare worker FGD involving 5 providers engaged in HIV and/or diabetes care. Qualitative data were analyzed using an inductive content analysis approach, and the emergent findings were interpreted through Burden of Treatment Theory. No participant took part in more than one data collection method.

Results: Participants described diabetes as adding a second layer of treatment work to an already-established HIV routine. Treatment burden was shaped by medication workload and regimen complexity, fragmented healthcare pathways, financial and time pressures, and cognitive and emotional strain. Mobile phones were used to help patients and supporters manage reminders, appointments, communication, and care coordination outside the clinic setting. Social support enhanced patient capacity by buffering these burdens through reminders, accompaniment, encouragement, and practical assistance. Mobile phones were already being used informally as reminder tools, communication channels, and care coordination resources; however, their effectiveness was constrained by shared device use, privacy concerns, airtime costs, charging difficulties, and low digital confidence. Participants preferred a mobile phone-based system that was integrated across HIV and diabetes care, discreet, low-cost, compatible with feature phones, and linked to trusted human support.

Conclusion: Medication-taking among adults living with HIV and type 2 diabetes was experienced as part of a broader treatment burden shaped by medication routines, clinic demands, financial constraints, food insecurity, privacy concerns, and social support needs. Mobile phones were already being used informally for reminders, appointment tracking, communication, and care coordination. A phone-based medication support intervention should therefore be low-burden, privacy-sensitive, linked to trusted support, and aligned with patients’ everyday phone practices.

## Introduction

The global burden of chronic diseases continues to rise, with type 2 diabetes and human immunodeficiency virus (HIV) representing two major and increasingly intersecting public health challenges, particularly in low- and middle-income countries (LMICs) [1-3]. Widespread access to potent antiretroviral therapy has transformed HIV into a chronic, manageable condition and substantially improved life expectancy, leading to a growing population of people ageing with HIV and accumulating non-communicable comorbidities such as diabetes, cardiovascular disease, renal disease, and depression [2].

In many LMICs, where diabetes prevalence is rising rapidly alongside persistent infectious disease burdens, the convergence of HIV and type 2 diabetes intensifies clinical, economic, and social pressures on patients, households, and health systems [1,2,4]. As people live longer with HIV and develop additional non-communicable conditions, the complexity of daily self-management and medication routines increases, particularly in resource-limited settings [5-7].

Managing HIV alongside type 2 diabetes requires sustained engagement with multiple and often competing treatment demands, including complex medication regimens, dietary adjustments, regular clinic visits, and continuous monitoring [8-10].

These demands impose a substantial treatment burden on patients, understood as the workload of healthcare and its impact on patient functioning and well-being [10-12]. Treatment burden is especially pronounced in multimorbidity, where individuals must juggle numerous health-related tasks alongside their social, emotional, and financial consequences [10,11].

In many LMICs, including several in sub-Saharan Africa, services for HIV and non-communicable diseases such as diabetes and hypertension are commonly delivered through separate vertical clinics. Patients with multiple conditions are therefore required to attend different services, often on different days and at different locations, while coordinating their own care across providers [9,13-15]. Patients report long waiting times, multiple appointments, conflicting advice, and fragmented records as major contributors to treatment workload and frustration [15].

Despite growing recognition of multimorbidity, most interventions designed to improve medication adherence remain disease-specific, typically targeting HIV or diabetes separately rather than addressing their combined burden. Evidence from LMICs shows that HIV adherence programs have focused mainly on antiretroviral therapy (ART) adherence through counselling, education, reminders (including short message service (SMS) and mobile health (mHealth) interventions), peer or community support, adherence clubs, and related supportive approaches [16-18]. In contrast, diabetes interventions have largely focused on glycemic control and cardiometabolic risk reduction through structured diabetes self-management education and lifestyle modification, including diet, physical activity, self-monitoring, and broader self-management support [19-22].

This siloed approach overlooks the cumulative and interacting treatment demands faced by people managing multiple chronic conditions, where polypharmacy, regimen complexity, and multiple appointments may undermine adherence and treatment outcomes [23-25].

Emerging research in multimorbidity and coexisting cardiometabolic conditions suggests that more multifaceted, patient-centered strategies integrating education, behavioral support, regimen simplification, and coordinated follow-up across conditions are needed to ensure that adherence interventions remain relevant and effective in real-world multimorbidity care [25,26].

mHealth technologies have emerged as promising tools for supporting medication adherence and chronic disease management in resource-constrained settings, where mobile phone access is high but health system capacity remains limited [27-30].

Recent evidence shows that mobile phone-based interventions, including SMS, interactive voice response (IVR) systems, and mobile applications, can improve ART adherence, clinic attendance, and, in some cases, viral suppression and retention in care among people living with HIV [27,31-33].

In Uganda, mHealth interventions have demonstrated high feasibility and acceptability for supporting ART adherence and patient engagement, including real-time electronic adherence monitors linked to SMS reminders and clinic-based use of electronic monitors with optional messaging in routine care [34-36].

However, their effectiveness depends heavily on sustained user engagement and on design features such as personalization, communication, reinforcement, usability, and compatibility with users’ everyday contexts and capacities, as engagement often declines over time and is rarely treated as a primary outcome [37-40].

At the same time, although interest in using digital health technologies for people living with multiple chronic conditions is rapidly growing, most available technologies, telemedicine platforms, and app-based interventions continue to be designed around single diseases. As a result, patients with multimorbidity are often required to manage multiple separate tools, while important contextual and care coordination challenges remain insufficiently addressed [41-44].

In everyday practice, patients commonly use mobile phones informally to support their health, for example, by setting medication and appointment reminders, communicating with family members, and coordinating care with health workers [45-48]. 

At the same time, social support networks, including family members, friends, peers, and healthcare providers, play a central role in promoting self-management, resilience, and medication adherence among people living with chronic conditions and multimorbidity [49-52]. 

Stronger social support has been associated with better coping, improved quality of life, and, in many contexts, higher adherence to treatment, although findings are not always consistent across settings [50,51,53,54].

However, how patients integrate mobile phone use with these offline and online support networks in the day-to-day management of multiple conditions remains poorly understood. Emerging research is only beginning to examine how social ties and digital tools jointly shape self-management practices [47,52,55,56]. 

Moreover, many digital and mHealth interventions demonstrate mixed or only short-term effects, partly because they are insufficiently grounded in patients’ lived experiences, social contexts, existing coping strategies, and support systems [57-59]. Integrating patient perspectives, local context, and social relationships into the design and adaptation of digital health tools is increasingly recognized as essential for achieving meaningful and sustained impact.

From an implementation science perspective, there is growing emphasis on designing interventions that are context-sensitive and minimally disruptive to patients’ everyday lives [60-62]. The Burden of Treatment Theory and related frameworks conceptualize treatment burden as the workload imposed by healthcare and its impact on well-being, particularly when healthcare demands exceed patients’ capacity, which is shaped by social, economic, cognitive, and physical resources [10,11].

Interventions that ignore treatment burden may overwhelm patients, contributing to non-adherence and disengagement. In contrast, approaches that reduce workload and enhance patient capacity, for example, through coordinated care, social support, simplified processes, and accessible technologies, are more likely to be acceptable, feasible, and sustainable in real-world multimorbidity care [10,11].

There is therefore a critical need for contextually grounded qualitative evidence examining how adults living with HIV and type 2 diabetes experience treatment burden in their everyday lives, how they informally use mobile phones and social support to manage this burden, and how these insights can inform the design of patient-centered phone-based medication support interventions. This study therefore aimed to (1) explore treatment burden among adults living with HIV and type 2 diabetes in Uganda; (2) examine how patients informally use mobile phones and social support to manage medication and care; and (3) identify user-informed requirements for a phone-based medication support intervention.

## Materials and methods

Theoretical framework

This study was informed by the Burden of Treatment Theory, which explains how patients experience and manage the workload created by healthcare demands in relation to their available capacity [62]. The main constructs of the theory are treatment workload, patient capacity, and the relationship between workload and capacity. Treatment workload refers to the tasks patients must perform to manage illness and care, including taking medications, attending clinic appointments, monitoring symptoms, following dietary advice, coordinating care, communicating with healthcare providers, and mobilizing support. Patient capacity refers to the resources patients can draw upon to manage this workload, including personal, social, financial, cognitive, emotional, and health-system resources. When treatment workload exceeds patient capacity, patients may experience treatment burden, which can negatively affect medication-taking, clinic attendance, emotional well-being, and engagement with care.

The theory informed both data collection and analysis. During data collection, the interview and focus group discussion (FGD) guides explored the work involved in managing HIV and type 2 diabetes, including medication routines, clinic attendance, food and transport demands, communication with health workers, social support, privacy concerns, and informal mobile phone use. Participants were also asked how phones and trusted supporters helped manage or complicated this work. During analysis, the theory guided the interpretation of how participants described treatment workload, which resources increased or reduced their capacity, and how mobile phones interacted with this balance. This approach enabled the study to interpret requirements for phone-based interventions not simply as preferred digital features, but as potential ways of reducing treatment workload, strengthening patient capacity, and supporting medication-taking in everyday life.

Study design and setting

This study employed a qualitative design using in-depth interviews and FGDs. A qualitative approach was appropriate because the study sought to explore lived experiences, meanings, support practices, informal mobile phone use, and user-informed intervention requirements among adults living with HIV and type 2 diabetes. In-depth interviews allowed participants to discuss personal and sensitive experiences in detail, including stigma, fear of disclosure, treatment fatigue, financial difficulties, and medication routines. FGDs enabled the study to capture shared experiences, collective perspectives, and practical considerations from patients, social supporters, and healthcare workers regarding treatment burden and future phone-based support interventions.

The study was conducted at Mbarara Regional Referral Hospital in southwestern Uganda. Participants were recruited from the hospital’s HIV and diabetes care clinics. Mbarara Regional Referral Hospital serves patients from Mbarara City and surrounding rural and peri-urban districts in southwestern Uganda. The setting was appropriate for this study because many patients living with HIV and type 2 diabetes receive long-term care at the hospital and must manage medication routines, clinic attendance, transport costs, food-related challenges, privacy concerns, and communication with healthcare workers within their everyday lives.

Study participants and sampling

Participants were purposively selected to capture diverse experiences and perspectives regarding HIV and type 2 diabetes medication management, treatment burden, social support, informal mobile phone use, and expectations for a future phone-based support system. The study included adults living with both HIV and type 2 diabetes, nominated social supporters, and healthcare workers involved in HIV and/or diabetes care at Mbarara Regional Referral Hospital.

A total of 64 unique participants contributed data. Of these, nine adults living with HIV and type 2 diabetes participated in individual in-depth interviews. The remaining 55 participants took part in 11 FGDs. These included six patient FGDs involving 30 adults living with HIV and type 2 diabetes, four social supporter FGDs involving 20 nominated supporters, and one healthcare worker FGD involving 5 providers engaged in HIV and/or diabetes care. Participants did not overlap across data collection methods; each participant contributed data through either an in-depth interview or a single FGD. Each FGD included approximately five participants.

Eligible patient participants were adults aged 18 years and above, living with both HIV and type 2 diabetes, receiving care at Mbarara Regional Referral Hospital, and willing to provide informed consent. Social supporters were adults identified by eligible patients as trusted individuals who provided day-to-day support, such as reminders, accompaniment, encouragement, assistance with clinic visits, financial support, or help with phone communication. Healthcare workers were eligible if they were involved in HIV care, diabetes care, counselling, pharmacy services, or related chronic care services at Mbarara Regional Referral Hospital.

Because HIV and diabetes services were delivered through separate clinic points, eligible patient participants were identified in collaboration with staff from both the HIV and diabetes clinics. Patients were considered eligible if they were receiving care at Mbarara Regional Referral Hospital and had both HIV and type 2 diabetes documented or recognized within their care pathway. Social supporters were nominated by eligible patients as trusted individuals involved in their day-to-day treatment routines, while healthcare workers were purposively selected based on their involvement in HIV care, diabetes care, counselling, pharmacy services, or chronic care follow-up.

Purposive sampling was appropriate because the study required participants with direct experience of living with HIV and type 2 diabetes, providing informal support, or delivering HIV and/or diabetes care, rather than a statistically representative sample.

Sampling continued alongside preliminary review of interview and focus group transcripts. Saturation was monitored during data collection through review of field notes and emerging issues after each set of interviews and FGDs. The lead author discussed emerging findings with the supervisory team to assess whether new information was still being generated. Saturation was considered to have been reached when later interviews and FGDs largely repeated issues identified in earlier data, particularly regarding medication workload, food and transport barriers, privacy concerns, social support, informal mobile phone use, and preferred features of a future phone-based support system. Towards the end of data collection, later interviews and FGDs provided additional illustrative detail but did not generate new major themes.

Data collection

Data were collected using semi-structured interviews and FGD guides. The guides explored participants’ experiences of living with HIV and type 2 diabetes, daily medication routines, treatment workload, clinic attendance, food- and transport-related challenges, emotional strain, stigma and privacy concerns, and the resources patients used to manage this workload, including social support and mobile phones. The guides also explored participants’ preferences for a future phone-based medication support intervention.

In-depth interviews were conducted with adults living with HIV and type 2 diabetes to obtain detailed personal accounts of managing both conditions. These interviews allowed participants to discuss sensitive issues privately, including stigma, fear of disclosure, treatment fatigue, financial difficulties, family support, and challenges in maintaining medication routines. FGDs were conducted separately with patients, social supporters, and healthcare workers to explore shared experiences, collective perspectives, and practical considerations for designing a phone-based support system.

Data collection was conducted by the lead author, a PhD researcher at Mbarara University of Science and Technology with training and experience in qualitative health research. The lead author conducted the in-depth interviews and moderated the FGDs, with support from trained research assistants where needed for note-taking, participant coordination, and field logistics. Interviews and FGDs were conducted in private rooms or quiet spaces within or near the clinic to ensure confidentiality and participant comfort. Patient FGDs were audio-recorded, conducted mainly in Runyankole/Rukiga with occasional English prompts, and held in private clinics or near-clinic spaces.

At the beginning of each session, participants were informed about the purpose of the study, the voluntary nature of participation, confidentiality, the use of audio-recording, and their right to decline any question or withdraw at any time without affecting their care. Open-ended questions and probes were used to allow participants to explain their experiences in their own words. Probing was particularly important for clarifying how treatment burden was experienced in daily life, how support was provided or limited, how phones were already being used, and which design features would make a future phone-based support system acceptable and practical.

The interview and FGD guides were developed based on the study objectives, Burden of Treatment Theory, and existing literature on multimorbidity, medication adherence, social support, and mobile phone use in chronic disease care. The guides included open-ended questions and probes related to daily medication routines, clinic attendance, food and transport challenges, emotional strain, privacy concerns, social support, informal mobile phone use, and preferred features of a future phone-based support system. Example questions included: “Can you describe how you manage your HIV and diabetes medicines in a normal day?”, “What makes it difficult to take your medicines or attend clinic?”, “How do you use a phone to support treatment or communicate about care?”, and “What kind of phone-based support would be useful and acceptable to you?” The guides were reviewed by the supervisory team for relevance, clarity, and sensitivity to the local context. Interviews and FGDs lasted approximately 45-90 minutes, depending on participant category, group size, and depth of discussion.

All interviews and FGDs were audio-recorded with participants’ permission. Field notes were used to capture contextual observations, group dynamics, non-verbal cues, and issues requiring follow-up during analysis. Basic participant information, including participant category, approximate age group, residence type, and role in care or support, was collected for descriptive purposes.

Data management

Audio-recordings were transcribed verbatim. Interviews and discussions conducted in Runyankole/Rukiga were translated into English by bilingual transcribers familiar with the local language and healthcare context. Transcripts were reviewed for completeness, clarity, and consistency with the audio-recordings. Identifying information, including names and other direct identifiers, was removed from all transcripts. Each transcript was assigned a study identifier reflecting the participant category and data collection method, such as patient in-depth interview, patient focus group discussion, social supporter FGD, or healthcare worker FGD.

All electronic files, including audio-recordings, transcripts, field notes, and analysis documents, were stored securely on password-protected devices accessible only to authorized members of the research team. Anonymized transcripts were used during coding, analysis, and manuscript development.

Data analysis

Data were analyzed using an inductive content analytic approach. This approach was appropriate because the study aimed to derive categories and themes directly from participants’ accounts while allowing interpretation through the Burden of Treatment Theory. Analysis began with repeated reading of transcripts to gain familiarity with the dataset and develop an overall understanding of participants’ experiences [63,64]. The lead author conducted manual open coding using Microsoft Word and Excel-based coding matrices (Microsoft Corp., Redmond, WA, USA). Coding focused on text segments related to medication routines, treatment workload, regimen complexity, clinic attendance, financial strain, food insecurity, transport difficulties, emotional burden, stigma, privacy, social support, phone access, informal mobile phone use, and preferences for a future phone-based support system.

Codes were compared across transcripts and grouped into broader categories. Similar codes were merged, overlapping codes were refined, and categories were organized around recurring patterns across the data [64]. A codebook was developed iteratively and included code names, definitions, inclusion and exclusion criteria, and illustrative quotations. The analysis moved back and forth between transcripts, coded data, categories, and emerging themes to ensure that the findings remained grounded in participants’ accounts. Saturation was assessed during analysis by comparing new transcripts with the developing codebook. Later transcripts added illustrative detail but did not generate new major categories.

To enhance analytic dependability, emerging codes, categories, and themes were discussed regularly with the supervisory team during analysis. The supervisory team reviewed selected coded extracts, emerging categories, and illustrative quotations to assess whether interpretations were grounded in the data and aligned with the study objectives. Differences in interpretation were resolved through discussion, returning to the transcripts, and refining code definitions and theme boundaries. Formal participant member checking was not conducted because of logistical constraints and the sensitivity of the study topic. However, credibility was strengthened through triangulation across patients, social supporters, and healthcare workers, as well as through comparison of findings across interviews and FGDs.

Following the inductive coding process, emerging themes were interpreted through the Burden of Treatment Theory by examining treatment workload, patient capacity, and the relationship between the two. This involved examining how participants described the workload associated with managing HIV and type 2 diabetes and how this workload interacted with their capacity to manage treatment in daily life. Particular attention was given to medication work, appointment work, food- and diet-related work, emotional work, coordination work, and communication work. The analysis also explored how social support and mobile phones either strengthened or constrained patients’ capacity to manage treatment demands.

Comparison across participant groups was used to strengthen interpretation. Patient interviews and FGDs were compared with discussions involving social supporters and healthcare workers to identify areas of agreement, difference, and practical relevance for intervention design. For example, patient accounts highlighted forgetting, food and transport challenges, privacy concerns, local language needs, and the importance of simple reminders. Social supporters emphasized consent, timing, and guidance on how to provide support without causing tension, while healthcare workers focused on feasibility, follow-up, and integration with routine care. These comparisons helped identify design requirements for a phone-based medication support intervention that would be acceptable, low-cost, privacy-sensitive, locally understandable, compatible with feature phones, and connected to trusted human support.

Trustworthiness and reflexivity

Several strategies were used to enhance the trustworthiness of the study. Credibility was strengthened through the inclusion of multiple participant groups, the use of both in-depth interviews and FGDs, and the use of open-ended questions and probes that allowed participants to explain their experiences in detail. Dependability was supported through the use of semi-structured guides, audio-recording, verbatim transcription, systematic coding, and iterative codebook development. Confirmability was enhanced by grounding the analysis in participants’ own accounts and retaining illustrative quotations during theme development. Transferability was supported by providing a clear description of the study setting, participant categories, sampling approach, data collection procedures, and contextual factors shaping treatment experiences and phone use.

Triangulation was achieved by comparing data across three participant groups: adults living with HIV and type 2 diabetes, social supporters, and healthcare workers. It was further strengthened through the use of both in-depth interviews and FGDs. Comparing these different data sources and methods helped identify areas of agreement, difference, and practical relevance for understanding treatment burden, informal phone use, social support, and future intervention requirements.

The research team remained attentive to reflexivity throughout data collection and analysis. Because the study involved sensitive issues such as HIV, diabetes, stigma, poverty, family relationships, and privacy, the interviewer emphasized voluntary participation, confidentiality, respect, and the absence of right or wrong answers. Participants were encouraged to speak in their own words and to raise issues that were important to them. This was particularly important because several intervention requirements, including local language, feature phone compatibility, voice-based options, trusted supporters, privacy-sensitive wording, and human follow-up, emerged strongly from participants’ own accounts.

Reflexivity was further addressed by considering the lead author’s position as a PhD researcher with an interest in digital health intervention development. During data collection and analysis, care was taken to avoid assuming that mobile phone support would automatically be acceptable or useful. Participants were encouraged to describe both positive and negative experiences related to phone use, social support, and healthcare communication. Field notes and analytic discussions were used to reflect on how the researcher’s role, study aims, and expectations regarding mHealth might influence probing, interpretation, and theme development.

Ethical considerations

Ethical approval was obtained from the Mbarara University Research Ethics Committee (REC No: MUST-2025-611), and regulatory clearance was obtained from the Uganda National Council for Science and Technology (Registration No: SIR624ES). Administrative permission was obtained from Mbarara Regional Referral Hospital prior to data collection. All participants provided informed consent before participating in the study. Participants were informed about the purpose of the study, study procedures, potential risks and benefits, confidentiality, voluntary participation, and their right to withdraw at any time without affecting their care.

Interviews and FGDs were conducted in private spaces to protect confidentiality. No real names were used in transcripts or reports. Given the sensitivity of HIV status and the possibility of unintended disclosure through phone communication, privacy was treated both as an ethical concern during data collection and as an important analytic issue in interpreting intervention requirements. Audio-recordings, transcripts, and analysis files were stored securely, and only anonymized quotations were used in reporting the findings.

## Results

Participant characteristics

A total of 64 unique participants took part in the study. Nine adults living with HIV and type 2 diabetes participated in individual in-depth interviews, while 55 participants participated in 11 FGDs. The FGDs included six patient FGDs with 30 adults living with HIV and type 2 diabetes, four social supporter FGDs with 20 nominated supporters, and one healthcare worker FGD with 5 providers involved in HIV and/or diabetes care. Each FGD had approximately five participants. No participant contributed data through more than one method. Patient participants were adults living with both HIV and type 2 diabetes and receiving care at Mbarara Regional Referral Hospital. Social supporters included spouses, adult children, relatives, friends, neighbors, and other trusted people who supported patients in daily treatment routines. Healthcare workers included providers involved in HIV and/or diabetes care, including nurses, clinical officers, counsellors, and pharmacy staff. The participants’ characteristics are described in Table 1.

**Table 1:** Participants’ characteristics FGD: focus group discussion, HIV: human immunodeficiency virus, IQR: interquartile range.

Baseline characteristic	Category	Participants, n (%)
Total participants		64 (100.0)
Data collection method	In-depth interviews	9 (14.1)
	Focus group discussions	55 (85.9); 11 sessions
Participant category	Adults living with HIV and type 2 diabetes	39 (60.9)
	Social supporters	20 (31.3)
	Healthcare workers	5 (7.8)
Patient data source	Patients in in-depth interviews	9
	Patients in focus group discussions	30
Focus group category	Patient FGDs	6 sessions; 30 participants; approximately 5 per FGD
	Social supporter FGDs	4 sessions; 20 participants
	Healthcare worker FGD	1 session; 5 participants
Approximate patient age	Age range	34-65 years
	Median age (IQR), patients only	50 (45-58)
Patient residence type	Rural	23 (58.9)
	Peri-urban	7 (17.9)
	Urban	9 (23.1)
Social supporter relationship to patient	Spouse/partner	3
	Adult child/granddaughter	4
	Sibling/relative/in-law	8
	Friend/neighbor/church/community supporter	5
Healthcare worker designation	HIV clinic nurse	1
	Clinical officer	1
	Diabetes clinic nurse	1
	Counsellor	1
	Pharmacist	1
Language	Runyankole/Rukiga	59 participants
	English	5 participants

Overview of themes

Across the interviews and FGDs, medication adherence was described as part of a wider treatment burden rather than as a matter of forgetfulness or personal discipline alone. Importantly, mobile phones were already woven into how patients, supporters, and healthcare workers managed parts of this burden, especially through reminders, appointment tracking, checking in, and communication outside the clinic. Five main themes were developed from the analysis: Theme 1, diabetes as an added layer of treatment work on an established HIV routine; Theme 2, the everyday workload of managing two chronic conditions; Theme 3, social support as both a source of capacity and a potential source of vulnerability; Theme 4, mobile phones as informal but limited tools for managing treatment burden; and Theme 5, user requirements for a phone-based medication support intervention. Theme 2 contained four sub-themes: financial burden, food insecurity, health-system burden, and emotional burden. Theme 4 contained three sub-themes: phones as everyday coordination tools, practical limits to phone-based support, and privacy as a central design concern. Therefore, the findings are presented under five main themes and seven sub-themes.

Theme 1: Diabetes as an Added Layer of Treatment Work on an Established HIV Routine

Participants commonly described HIV treatment as something they had gradually learned to live with, while diabetes was experienced as a newer and more disruptive addition. Many patients reported that they had already developed a routine for antiretroviral therapy, clinic attendance, and living with HIV. However, when diabetes was diagnosed, it introduced additional medication, dietary expectations, bodily symptoms, and clinic-related demands. This was experienced not only as having “another disease,” but as having another layer of daily work added to an already demanding life.

Patients explained that diabetes required more attention to food, timing, weakness, dizziness, and body changes. Unlike HIV treatment, which many had internalized over time, diabetes care felt less settled and more difficult to fit into daily routines. For some participants, this made diabetes medication easier to forget or delay, especially when the day became busy, when food was not available, or when the patient was away from home.

“HIV had already become part of my life because I had learned how to take treatment well. But when diabetes came, it felt like another burden had been added. Now every day I must think about many things at once: medicine, food, clinic appointments, weakness in the body, and even stress.” (Patient FGD participant)

Another patient similarly described the mental burden of managing both conditions:

“Before, I only worried about one line of medicine. Now there is food, sugar, clinic, and body weakness. It is a lot.” (Patient FGD participant)

Healthcare workers confirmed this pattern. They observed that many patients had relatively stronger ART routines because they had been in HIV care for longer, while diabetes medication taking was often more difficult to sustain because diabetes care required additional counselling around food, symptoms, timing, and self-monitoring. One healthcare worker explained that dual-condition patients were not simply managing two diagnoses, but were trying to fit into two different care routines and expectations.

“In the HIV clinic, many patients have already developed a fairly good routine for ART because they have been in care for long. But once diabetes is added, the pattern changes. Diabetes medicines often require more discussion around food, timing, symptoms, and self-monitoring.” (HIV clinic nurse)

This theme suggests that medication taking among adults living with HIV and type 2 diabetes should be understood within the broader experience of treatment accumulation. Diabetes did not replace HIV care; rather, it added a second, less familiar and more demanding routine that patients had to manage alongside existing HIV treatment.

Theme 2: The Everyday Workload of Managing Two Chronic Conditions

Participants described treatment burden as extending beyond medicine-taking. The workload of living with HIV and type 2 diabetes involved securing transport, finding suitable food, attending clinic reviews, managing body weakness, protecting privacy, and coping with worry. These demands interacted with poverty, household responsibilities, work, distance from the hospital, and uncertainty about symptoms. Four sub-themes captured this workload: financial burden, food insecurity, health-system burden, and emotional burden.

Financial burden: Financial strain shaped how participants managed both conditions. Transport costs were repeatedly described as a barrier to clinic attendance, especially for participants from rural areas. Review days created anxiety when patients lacked money for travel, and missed appointments could delay medicine refills and follow-up. Financial burden was therefore not separate from adherence; it directly affected whether patients could reach the clinic, collect medicines, and maintain routine care.

“If review day comes when there is no transport, you start struggling.” (Patient FGD participant)

For some participants, poverty also affected decisions around medicine-taking. Patients understood the importance of medicines, but financial hardship made it difficult to meet the conditions needed for safe and consistent use, especially where food and transport were both uncertain.

Food insecurity: Food insecurity was one of the clearest ways in which diabetes increased treatment workload. Participants explained that diabetes care required attention to food type and timing, but household realities often made dietary advice difficult to follow. Patients were sometimes told to avoid certain foods or eat at particular times, yet they had to eat what was available.

“You may be told to avoid certain foods or to eat at the right time, but you eat what is available. So it’s sometimes hard to follow the doctor’s advice.” (Patient FGD participant)

Food insecurity also affected confidence in taking diabetes medicines. Some participants feared taking medicines when they had not eaten, because they associated this with weakness, dizziness, or feeling unwell. This made medication adherence dependent not only on memory, but also on whether patients could access food at the right time.

Health-system burden: Participants also described the workload created by clinic attendance and care coordination. Managing both HIV and type 2 diabetes required keeping track of appointments, attending reviews, obtaining refills, and understanding advice from different care points. Missing clinic was not experienced as a single event; it could interrupt medicine supply, increase worry, and weaken confidence in the treatment routine.

Healthcare workers also recognized that adherence challenges were not only clinical. They described patients as trying to follow treatment instructions within social and economic conditions that often made those instructions difficult to implement.

“Some are tired of swallowing many tablets. Some are confused about which medicine is for what. Others know what to do but fail because their social or economic reality does not support what we advise. So the challenge is not only clinical. It is behavioural, social, and structural at the same time.” (Diabetes clinic nurse)

This shows that the health system contributed to treatment burden when patients had to manage multiple appointments, medicines, and instructions without enough integrated support.

Emotional burden: The burden of living with both conditions was also emotional. Participants described worry, mental tiredness, fear of symptoms, and discouragement when routines were disrupted. Some were uncertain whether weakness or dizziness came from diabetes, HIV, hunger, or medicines. This uncertainty increased anxiety and made patients feel that their lives were constantly organized around illness.

“Sometimes the challenge is not that you do not want treatment. The problem is daily life. Life itself pushes you away from the routine.” (Patient FGD participant)

Emotional burden affected adherence by reducing confidence and peace of mind. Once routines were disrupted, some participants felt they were failing themselves, even when the reasons were linked to poverty, food, transport, or family demands. Medication adherence was therefore embedded in the practical and emotional realities of daily life.

Theme 3: Social Support as a Source of Capacity and Vulnerability

Social support was described as an important resource for managing treatment burden. Patients received support from spouses, children, siblings, friends, fellow patients, and healthcare workers. This support included medication reminders, appointment reminders, reading messages, accompaniment, encouragement, help with transport, and emotional reassurance. For many participants, reminders from trusted people helped restore routine when daily life became disorganized.

“For me my wife helps a lot. She reminds me, especially when she notices I am late. Even when she just asks, ‘Have you swallowed?’ it helps me return to the routine.” (Patient FGD participant)

Support was most valued when it was respectful, private, and connected to real-life needs. Participants emphasized that reminders should not be harsh or embarrassing. Some described support as helpful only when it recognized the practical barriers patients faced, such as lack of food, lack of transport, or emotional tiredness. Encouragement was important because patients sometimes felt overwhelmed by the demands of two lifelong conditions.

“Reminders and encouragement are the biggest. But practical support also matters. Even just helping someone get transport or food can support treatment.” (Patient FGD participant)

However, social support was not always sufficient or easy to use. Some family members were willing to help but were also struggling with their own responsibilities. Others were absent, busy, or lacked knowledge about how to support a person managing both HIV and diabetes. Privacy and stigma also limited how much support patients could accept. Some patients feared disclosing too much, especially about HIV, and therefore avoided involving people who might otherwise have helped them.

“There are also people who fear to tell others in full because of stigma. So support may be there around you, but you cannot use it fully because you want privacy.” (Patient FGD participant)

Social supporters also described their role as important but sometimes difficult. They wanted to help without exposing the patient, causing conflict, or being seen as controlling. They also expressed a need for guidance on how to support patients properly, especially when medicines, clinic appointments, diet, and emotional distress had to be managed together.

“I would want a simple system that helps us support without exposing the person.” (Social supporter FGD participant)

This shows that social support increased patients’ capacity to manage treatment burden, but it could also introduce new sensitivities around privacy, relationship dynamics, and supporter workload. A phone-based intervention would therefore need to strengthen support without increasing stigma, surveillance, or conflict within households.

Theme 4: Mobile Phones as Informal but Limited Tools for Managing Treatment Burden

Participants were already using mobile phones in informal ways to manage treatment and care. Patients used phones to call family members, ask about clinic dates, communicate with trusted people, receive occasional clinic reminders, and seek help when unwell. Social supporters used calls, short messages, WhatsApp, and voice notes to check on patients, encourage them, and remind them about medicines or appointments. Healthcare workers also reported using phones informally to follow up patients who missed appointments, clarify concerns, and support patients after counselling or pharmacy encounters.

These phone practices show that digital support was already part of patients’ treatment routines, although it was informal, uneven, and dependent on individual relationships. Phones helped distribute treatment work across patients, supporters, and healthcare workers, but they also introduced new concerns around cost, access, privacy, and reliability. Three sub-themes captured this pattern: phones as everyday coordination tools, practical limits to phone-based support, and privacy as a central design concern.

Phones as everyday coordination tools: Patients, supporters, and healthcare workers described phones as ordinary tools for coordinating care. Patients used phones to call family members, ask about clinic dates, receive occasional reminders, and seek help when unwell. Social supporters used calls, SMS, WhatsApp, and voice notes where possible to check on patients and remind them about medicines or appointments. Healthcare workers also reported using phone calls informally to follow up patients who had missed appointments or needed clarification.

“Mostly for calling family members or asking about clinic days.” (Patient FGD participant)

“We sometimes call patients who have missed appointments or who need clarification. Some reminders are also passed through phones, but it is not yet a structured system.” (HIV clinic nurse)

This shows that phones were already part of the care environment, but not yet organized into a reliable support system.

Practical limits to phone-based support: Although phones were useful, participants described several limits. Some patients used feature phones and mainly relied on calls and SMS. Others had difficulty reading messages, especially when messages were long or written in English. Airtime costs, Internet bundles, poor network, lack of electricity, and battery charging problems also affected whether phones could be used consistently.

“Mine is a simple phone, so I mainly use calls and sometimes SMS.” (Patient FGD participant)

“Messages can help, but language matters. If they come in English, not everyone understands well.” (Patient FGD participant)

These limits show that a future phone-based intervention must not assume smartphone ownership, constant connectivity, strong literacy, personal phone ownership, or regular access to electricity. Simpler channels such as local-language SMS, calls, or voice options may be more realistic for this population. The findings also indicate that patients with no personal phone access or limited confidence in using phones would require alternative forms of support, such as assistance from a trusted supporter, clinic-based follow-up, or voice-based communication rather than text-heavy digital tools.

Privacy as a central design concern: Privacy was the strongest concern across participant groups. Patients feared that messages mentioning HIV, diabetes, medicines, or clinic attendance could be seen by other people, especially where phones were shared or handled by family members. Participants therefore preferred discreet wording that would remind them without exposing their health status.

“Privacy is the biggest concern. Messages should not openly mention HIV.” (Patient FGD participant)

Healthcare workers shared this concern and emphasized that phone-based support should not create new risks of disclosure.

“Privacy is the first concern. Messages must be discreet. If a notification openly mentions HIV, it can expose the patient.” (Pharmacist)

Theme 5: User Requirements for a Phone-Based Medication Support Intervention

Participants described a future phone-based system as potentially useful if it directly addressed the realities of living with both HIV and type 2 diabetes. Their requirements were shaped by both the treatment burden they experienced and the limits of their current informal phone use. The most frequently preferred features were medication reminders, appointment reminders, simple educational messages, encouraging messages, direct contact with a health worker, and optional involvement of a trusted supporter.

Medication reminders were prioritized because forgetting or delaying diabetes medication was common, especially when routines were disrupted by work, travel, food insecurity, stress, or family responsibilities. Appointment reminders were also considered important because missing clinic could interrupt medicine refills and follow-up.

“Medication reminders would help me most because forgetting is a real problem.” (Patient FGD participant)

“Appointment reminders also high, because if you miss clinic, even medicine refills become a problem.” (Patient FGD participant)

Participants wanted educational content, but only if it was short, practical, and in a language they understood. They preferred messages about food, medicine timing, living with both conditions, what to do when feeling weak, and why adherence matters. They did not want long, technical, or frequent messages that could become tiring.

“It should also send simple educational messages in our language, not complicated English.” (Patient FGD participant)

“Long messages may not work well. People will stop reading.” (Patient FGD participant)

Encouraging messages were also valued. Participants wanted support that was gentle, respectful, and non-judgmental, especially when they missed a dose or clinic appointment. Social supporters similarly emphasized that a system should keep care human and hopeful, rather than making patients feel monitored or blamed.

“Let it not judge people. Sometimes when one misses, they are already feeling bad. The support should bring them back kindly.” (Patient FGD participant)

The optional involvement of a trusted supporter was seen as useful, but only with patient consent. Participants emphasized that not everyone in the household should know the patient’s health issues. A supporter could receive reminders, help with clinic dates, encourage the patient, or know when to check more closely, but this had to be handled privately and respectfully.

“I would also like it to involve one trusted person at home, but only with my permission. Not everyone should know my issues.” (Patient FGD participant)

Healthcare workers emphasized that the system should connect patients, supporters, and clinics without creating parallel work. They preferred a system that could help identify patient needs, reinforce medication messages, support follow-up, and alert providers when human input was necessary.

“The system should support clinical decision-making rather than create parallel work. That is the balance.” (Clinical officer)

Participants also emphasized simplicity, low cost, compatibility with feature phones, local language, privacy-sensitive wording, flexibility in timing, and integration with routine care. Many were clear that a system requiring smartphones, Internet bundles, constant staff response, heavy reading, or advanced phone skills would exclude many patients, especially those with no personal phone access, shared phones, or low confidence in using mobile phone functions.

“Keep it simple. If it is too complicated, many will not use it.” (Patient FGD participant)

“Let it work on ordinary phones, not only smartphones.” (Patient FGD participant)

“Simplicity will matter more than sophistication.” (Pharmacist)

Participants wanted a system that reduced treatment burden, strengthened existing social and clinical support, protected privacy, and fitted the ordinary realities of patients’ lives. The preferred intervention was therefore one that combined discreet medication and appointment reminders, local-language education, encouragement, optional supporter involvement, and a realistic pathway for linking patients to health workers when additional human support was needed.

These findings point to five core design requirements for a future phone-based medication support intervention. These requirements emerged from the interaction between treatment burden and existing informal phone practices. First, the system should support HIV and diabetes care together, because patients experience both conditions as part of one daily routine. Second, it should reduce treatment workload through timely reminders, simple guidance, and practical support cues. Third, it should allow patient-controlled involvement of trusted supporters. Fourth, communication should be discreet, private, and available in a language patients understand. Finally, the system should work on feature phones that support calls and SMS as well as smart phones and remain linked to human support, because participants valued phones as tools for strengthening, not replacing, relationships with family members, supporters, and healthcare workers.

## Discussion

Principal findings

Guided by the Burden of Treatment Theory [62], the findings show how treatment workload, patient capacity, and the fit between the two shaped medication-taking and phone-based support needs among adults living with HIV and type 2 diabetes. Participants did not describe adherence simply as a matter of remembering medicines. Rather, they described a daily struggle shaped by multiple medicines, clinic appointments, food and transport needs, emotional strain, privacy concerns, and the need to depend on others for support. Diabetes was experienced as an added layer of work on top of an HIV routine that many patients had already learned to manage. A key digital health finding is that patients and supporters were already using mobile phones to manage parts of this workload through reminders, calls, saved dates, checking in, and communication with healthcare workers. Phone use therefore interacted directly with treatment burden by helping patients organize, share, and respond to treatment demands outside the clinic.

Social support played an important role in helping patients manage this burden. Supporters reminded patients to take medicines, accompanied them to the clinic, helped with transport or food, offered encouragement, and assisted with communication. However, support was useful only when it was respectful, trusted, private, and sensitive to the patient’s situation. Mobile phones were already being used informally to manage medicines, clinic appointments, and communication with supporters and healthcare workers. However, their usefulness was limited by shared phone use, privacy concerns, airtime costs, charging problems, poor network, language barriers, and limited confidence with more advanced phone functions.

Participants preferred a future phone-based medication support intervention that was simple, discreet, affordable, understandable, compatible with basic phones, and connected to trusted human support. These findings suggest that a phone-based intervention for this population should not be designed as a stand-alone reminder tool. It should be designed as a practical support system that reduces the burden of treatment, strengthens existing support relationships, and fits into patients’ daily lives.

Strengths of the study

This study provides a contextually grounded account of treatment burden among adults living with both HIV and type 2 diabetes in southwestern Uganda. One strength of the study is that it included patients, social supporters, and healthcare workers. This allowed medication management and treatment burden to be understood from different but connected perspectives. Patients described the daily work of managing both conditions, supporters explained the practical and emotional roles they play, and healthcare workers highlighted the realities of providing care within busy clinical settings.

Another strength is the use of both in-depth interviews and FGDs. The in-depth interviews allowed patients to discuss personal experiences that may not easily come out in a group setting, while the FGDs helped capture shared concerns, common support practices, and collective views about phone-based support. This combination strengthened the depth and range of the findings.

The use of the Burden of Treatment Theory also strengthened the analysis. It helped interpret the findings beyond individual behavior and showed how adherence is shaped by the balance between healthcare workload and patient capacity. This is important for intervention design because it shifts attention from simply asking patients to remember their medicines to designing support that makes treatment easier to manage.

Limitations of the study

The findings should be interpreted with some limitations in mind. First, the study was conducted at Mbarara Regional Referral Hospital in southwestern Uganda. Although participants came from rural, peri-urban, and urban settings, the findings may not fully represent the experiences of patients receiving care from smaller health facilities, private clinics, or other regions of Uganda. The findings are therefore more useful for conceptual and practical transferability than for statistical generalization.

Second, some participant characteristics, such as exact sex distribution, duration of living with HIV and type 2 diabetes, education level, income, and type of phone owned, were not recorded consistently across all transcripts. This limited the amount of demographic detail that could be presented in the manuscript. Third, because this was a qualitative study, the findings describe experiences, meanings, support practices, and intervention preferences rather than measuring the size of the problem or testing the effect of an intervention.

Fourth, coding and initial theme development were conducted primarily by the lead author. Although this allowed close engagement with the data, it may have introduced the possibility of single-researcher interpretation. To reduce this limitation, emerging codes, categories, themes, and illustrative quotations were discussed with the supervisory team, and interpretations were checked against the transcripts during analysis. However, formal independent double-coding was not conducted. Future qualitative work could include independent coding by more than one researcher or formal inter-coder comparison to further strengthen analytic dependability.

Finally, some participants may have been cautious when discussing sensitive issues such as missed medicines, stigma, poverty, household tensions, or fear of disclosure. However, the use of private interview spaces, separate participant groups, confidentiality assurances, and open-ended probing helped participants speak freely and provide rich accounts of their experiences.

Meaning of the study and comparison with other studies

The findings show that medication-taking among adults living with HIV and type 2 diabetes cannot be fully understood as an individual act of remembering or forgetting. It is part of a broader treatment burden shaped by the health system and by the resources available in everyday life. This is consistent with the Burden of Treatment Theory, which explains that patients with chronic illness carry a workload that includes medicines, appointments, monitoring, lifestyle adjustments, communication with providers, and coordination of care [62]. Similar treatment-burden literature has also shown that this workload becomes heavier when patients have more than one chronic condition and must manage the practical, emotional, and social consequences of care [10-12].

These interpretations should be understood within the context of this qualitative formative study conducted among adults living with HIV and type 2 diabetes, social supporters, and healthcare workers at Mbarara Regional Referral Hospital in southwestern Uganda. The findings are not intended to be statistically generalizable to all patients with HIV and type 2 diabetes in Uganda or other settings. Rather, they provide context-specific insights that may inform the design of phone-based medication support interventions in similar low-resource, multimorbidity care contexts.

Our findings agree with previous studies on HIV and non-communicable disease care in sub-Saharan Africa, which show that people with multimorbidity often carry the burden of coordinating care across services that remain largely organized around single diseases [9,65,66]. In the present study, diabetes disrupted an HIV treatment routine that many patients had already accepted and organized around. For several participants, HIV medicines and clinic visits had become familiar over time, whereas diabetes introduced new demands around food, symptoms, weakness, timing of medicines, and clinic attendance. Unlike studies that focus mainly on HIV adherence or diabetes self-management as separate conditions, this study shows how the two conditions interact in everyday life and create a combined treatment workload for patients.

Our findings are also consistent with previous work showing that patient capacity is shaped not only by individual motivation, but also by family, social, and healthcare relationships [9,23,65]. In this study, support from spouses, children, relatives, friends, fellow patients, and healthcare workers helped patients maintain routines, attend clinic, understand messages, and cope emotionally. However, our findings extend this literature by showing that social support can also create vulnerability when privacy, disclosure, stigma, and household relationships are not carefully managed. Therefore, future interventions should not assume that involving a supporter is always acceptable; supporter involvement must be voluntary, trusted, and guided by the patient.

Our findings agree with Ugandan studies showing that phone-based systems, including SMS reminders, electronic adherence monitoring, and IVR-supported ART adherence, can be feasible and acceptable when they are simple, responsive to user needs, and sensitive to privacy and local context [34,67-69]. However, the present study extends this evidence by showing that adults living with both HIV and type 2 diabetes require phone-based support that addresses multimorbidity rather than a single disease. Unlike many disease-specific mHealth interventions, participants in this study preferred support that recognized HIV and diabetes together, protected privacy, worked on ordinary phones, and remained connected to trusted human support.

Ugandan studies of real-time adherence monitoring, SMS reminders, electronic adherence monitors, and IVR-supported ART adherence have shown that phone-based systems can be feasible and acceptable when they are simple, responsive to user needs, and sensitive to privacy and local context [34,67-69].

The present study extends this work to adults living with both HIV and type 2 diabetes by showing that phone-based support must be designed for multimorbidity, where the same patient is managing more than one treatment routine, more than one clinic relationship, and multiple sources of social and financial pressure.

However, our findings differ from approaches that treat phone ownership or phone access as sufficient evidence of digital health readiness. In this study, phone-based support was shaped by shared phone use, low literacy, language preferences, airtime costs, charging problems, network challenges, and fear of disclosure. This is consistent with minimally disruptive care, which argues that healthcare should fit the patient’s life and capacity rather than adding unnecessary work [70,71]. Therefore, for this population, a useful phone-based intervention would need to reduce the work of being a patient rather than add another task to an already heavy routine.

The findings also highlight the digital divide in a practical and local form. In this study, the divide was not only about whether a participant owned a phone. It was also about whether the phone was shared, whether it could be charged, whether the patient had airtime or data, whether messages could be read and understood, whether network coverage was reliable, and whether phone use could happen without exposing sensitive health information. This means that future phone-based interventions should not assume that mobile phone access automatically translates into usable digital health access. For this population, equity in digital health design means building for basic phones, local language, low literacy, low cost, intermittent connectivity, and shared household phone use.

From a digital health perspective, these findings show that intervention design should begin from patients’ existing phone practices rather than from assumptions about advanced technology use. Participants were already using basic phone functions such as calls, SMS, saved dates, alarms, and WhatsApp, where possible. These practices were simple but important because they helped patients distribute treatment work across time, household relationships, and clinic contact.

The design contribution of this study is therefore not simply that patients need reminders. The findings show that patients need support that reflects how treatment is actually lived: HIV and diabetes together, food and medicine together, clinic and transport together, privacy and support together, and phones and human relationships together. A phone-based intervention that is technically functional but insensitive to these realities may increase burden instead of reducing it.

Implications for digital health design: proposed design framework

The findings point to a set of design principles for developing a phone-based medication support intervention for adults living with HIV and type 2 diabetes. These principles are grounded in participants’ everyday phone practices, treatment workload, social support arrangements, and concerns about privacy, cost, literacy, and access. The framework emphasizes that a useful phone-based intervention should not only remind patients to take medicines, but should also reduce the work of managing two chronic conditions, protect confidentiality, and strengthen trusted human support (Table 2).

**Table 2:** Design framework for a phone-based medication support intervention for adults living with HIV and type 2 diabetes In summary, these design principles suggest that the intervention should be understood as a digitally enabled support system rather than a simple reminder tool. Its value lies in connecting patients, selected supporters, and healthcare workers in ways that reduce treatment workload while protecting privacy. For adults living with HIV and type 2 diabetes, the design challenge is not only to deliver messages, but to ensure that phone-based support fits the social, economic, linguistic, and clinical realities in which medication adherence takes place. HIV: human immunodeficiency virus, SMS: short message service.

Design principle	Rationale	Key design features
Privacy and discretion	Privacy concerns, especially around HIV, were the most consistently reported barrier to phone-based support. Shared phone use increased the risk that reminders or messages could be seen by others.	Use neutral, non-disease-specific wording; allow users to control message content and timing; allow notifications to be disabled or customized; avoid explicit mention of HIV, diabetes, clinic, or medicines unless chosen by the patient; use wording such as "It is time for your routine" instead of "Take your HIV medication."
Integrated support for HIV and diabetes care	Participants experienced HIV and diabetes together in daily life, yet care routines were often fragmented. A disease-specific tool may increase rather than reduce workload.	Provide combined medication and appointment support for both conditions; allow appointment alignment reminders; include brief guidance that recognizes both HIV and diabetes care; avoid separate systems that force patients to manage multiple digital tools.
Low-burden and basic-phone compatibility	Many participants used ordinary phones or had limited confidence with smartphones. Airtime costs, charging problems, network challenges, and literacy affected phone use.	Design for SMS and voice calls, not only smartphone applications; use short messages; provide local-language options; include voice support for low-literacy users; minimize dependence on internet bundles; keep interaction steps few and simple.
Patient-controlled social support	Social supporters helped with reminders, encouragement, transport, food, and emotional support, but supporter involvement could also raise concerns about disclosure, control, and household tension.	Allow patients to choose whether to involve a supporter; let patients select who receives reminders; define what information the supporter receives; allow the patient to change or remove a supporter; include guidance for supporters on respectful and non-judgmental support.
Human-centered support, not technology alone	Participants valued phone reminders but also wanted access to trusted people when they were confused, unwell, discouraged, or facing treatment difficulties.	Include an option to contact a healthcare worker or clinic contact person; provide escalation for missed appointments or repeated non-response; combine automated reminders with human follow-up where needed; avoid designing the system as a purely automated reminder tool.
Local language and understandable communication	Participants emphasized that English messages and long texts may not be understood by all patients, especially older adults or those with lower literacy.	Provide Runyankole/Rukiga and other local-language options; use short, familiar, respectful wording; avoid technical medical language; allow voice messages or calls where reading is difficult.
Affordability and sustainability	Airtime, data costs, and transport-related poverty shaped whether patients could use phone-based support consistently. A system that is costly to use may exclude the patients who need it most.	Use low-cost channels such as SMS, missed-call options, or clinic-supported calls; minimize patient-paid data requirements; consider toll-free or reverse-billed communication; design for routine clinic use without heavy additional workload.
Flexible timing and personalization	Treatment routines differed by patient, medicine schedule, work, food availability, and household responsibilities. Fixed reminders may be ignored or become intrusive.	Allow patients to choose reminder times; permit adjustment of frequency; include appointment reminders in advance; allow temporary pausing or changing of reminders; tailor support to the patient's medicine and clinic routine.
Fit within clinic workflow	Healthcare workers supported phone-based follow-up but were concerned about creating parallel work in already busy clinics.	Link the system to existing HIV and diabetes clinic processes; define clear roles for health workers; use automated reminders for routine support and human response only when needed; generate simple follow-up lists rather than complex dashboards.

This framework is also consistent with digital health and mHealth evidence, showing that interventions are more likely to be acceptable and sustained when they are simple, interactive where needed, tailored to the user’s context, and integrated into existing care relationships rather than delivered as stand-alone technologies. In this study, participants did not ask for a complex application. They described the need for a low-burden system that works with ordinary phones, supports both HIV and diabetes care, uses discreet and locally understandable communication, allows patient-selected supporter involvement, and provides a way to reach a health worker when human support is needed.

Recommendations and future research

Based on these findings, future work should focus on co-developing and piloting a low-burden, privacy-sensitive, feature-phone-compatible medication support intervention with adults living with HIV and type 2 diabetes, social supporters, and healthcare workers. Such an intervention should integrate support for both HIV and diabetes care, use discreet and locally understandable communication, allow patient-controlled involvement of trusted supporters, and remain linked to human support within routine clinic workflows. Future studies should also examine how these experiences and intervention needs vary by gender, age, literacy level, residence, phone ownership, type of phone, duration of living with HIV and type 2 diabetes, and level of available social support.

Future studies should also explore how patients decide whether to involve a social supporter in medication support. Although many participants valued reminders, encouragement, and practical help from trusted people, others were cautious because of stigma, fear of disclosure, household tensions, or the desire to remain independent. Understanding how patients choose supporters, what information they are willing to share, and how supporter involvement should be managed is important for designing support systems that strengthen care without creating new risks.

The next phase of this work should focus on co-developing and piloting a phone-based medication support intervention with patients, social supporters, and healthcare workers. This intervention should be assessed for acceptability, feasibility, usability, privacy, cost, and preliminary implementation outcomes within routine HIV and diabetes care. Future implementation studies should assess the acceptability, feasibility, usability, privacy, cost, and preliminary implementation outcomes of such a system within routine HIV and diabetes care. They should also examine how the system can be integrated into clinic workflows without increasing healthcare-worker workload, and whether it can improve medication-taking, appointment keeping, patient confidence, supporter engagement, and patient-reported treatment burden over time.

## Conclusions

Guided by the Burden of Treatment Theory, this study showed that adults living with HIV and type 2 diabetes in southwestern Uganda experience medication management as part of a broader treatment burden. This burden includes medication complexity, fragmented care, financial strain, food-related challenges, emotional work, privacy concerns, and the need to mobilize support from others. Mobile phones are already being used informally to support medication-taking, appointment tracking, communication with supporters, and contact with healthcare workers although their use remains constrained by practical, social, and technological barriers. This shows that phone-based support is most promising when it is designed around how patients already use phones to manage treatment demands, rather than around technology alone.

A future phone-based medication support intervention should therefore be integrated across HIV and diabetes care, discreet, simple, low-cost, locally understandable, compatible with feature phones and smart phones, and linked to trusted human support. Because this was a qualitative formative study conducted in one referral hospital setting, these conclusions should be interpreted as context-specific design implications rather than evidence of intervention effectiveness or broad generalizability. The design framework proposed in this paper therefore positions privacy, integrated multimorbidity support, feature-phone compatibility, patient-selected social support, and access to human follow-up as core principles for future phone-based medication support. Such an intervention is more likely to strengthen adherence if it reduces the work of being a patient and fits the realities of everyday life.
